# Species-Specific Interferon-Gamma Release Assay for the Diagnosis of *Mycobacterium abscessus* Complex Infection

**DOI:** 10.3389/fmicb.2021.692395

**Published:** 2021-07-12

**Authors:** Mathis Steindor, Florian Stehling, Margarete Olivier, Jan Kehrmann, Margo Diricks, Florian P. Maurer, Peter A. Horn, Svenja Straßburg, Matthias Welsner, Sivagurunathan Sutharsan, Monika Lindemann

**Affiliations:** ^1^Pediatric Pulmonology and Sleep Medicine, Children’s University Hospital Essen, University of Duisburg-Essen, Essen, Germany; ^2^Institute of Medical Microbiology, University Hospital Essen, University of Duisburg-Essen, Essen, Germany; ^3^Molecular and Experimental Mycobacteriology, Research Center Borstel – Leibniz Lung Center, Borstel, Germany; ^4^National and WHO Supranational Reference Center for Mycobacteria, Research Center Borstel, Borstel, Germany; ^5^Institute of Medical Microbiology, Virology and Hygiene, University Medical Center Hamburg-Eppendorf, Hamburg, Germany; ^6^Institute for Transfusion Medicine, University Hospital Essen, University of Duisburg-Essen, Essen, Germany; ^7^Department of Pulmonary Medicine, Adult Cystic Fibrosis Center, University Hospital Essen – Ruhrlandklinik, University of Duisburg-Essen, Essen, Germany

**Keywords:** *Mycobacterium abscessus*, cystic fibrosis, non-tuberculous mycobacteria, pulmonary infection, immune response

## Abstract

*Mycobacterium abscessus* complex (MABC) infection has a devastating impact on the course of cystic fibrosis (CF) and non-CF lung disease. Diagnosis of MABC pulmonary disease is challenging, and current diagnostic approaches lack accuracy, especially in CF. In this study, we aimed to establish an MABC-specific interferon-γ release assay to detect host immune responses to MABC and improve diagnostics of MABC infection by the detection of antigen-specific T cells. Four species-specific proteins of MABC were overexpressed in an *Escherichia coli* expression system. Purified proteins were used to stimulate peripheral blood mononuclear cells of study subjects in an ELISpot assay. Interferon-γ response of 12 subjects with established diagnosis of MABC infection (10 CF and two non-CF) was compared with 35 controls (22 CF and 13 non-CF) distributed to three control groups, 17 CF subjects without NTM infection, nine subjects with NTM infection other than MABC, and nine subjects with tuberculosis. Cellular *in vitro* responses in the MABC group were stronger than in the control groups, especially toward the protein MAB_0405c (39 vs. 4 spots per 300,000 PBMC, *p* = 0.004; data represent mean values) in all patients and also in the subgroup of CF subjects (39 spots vs. 1 spot, *p* = 0.003). Receiver operating characteristic curve analysis indicated that spot numbers of at least 20 were highly predictive of MABC infection (all patients: area under curve 0.773, sensitivity 58%, and specificity 94%; CF patients: area under curve 0.818, sensitivity 60%, and specificity 100%). In conclusion, we identified MAB_0405c as a protein that may stimulate MABC-specific interferon-γ secretion and may add to the diagnosis of MABC infection in affected patients.

## Introduction

Members of the *Mycobacterium abscessus* complex (MABC) are rapidly growing mycobacteria causing lung infection predominantly in patients with preexisting lung diseases with often devastating impact (Park and Olivier, 2015). MABC is the most frequently isolated species of non-tuberculous mycobacteria (NTM) in Caucasian CF populations (Hill et al., 2012) and the second largest group of NTM in non-CF lung disease patients, after the *Mycobacterium avium* complex (Prevots et al., 2010). Compared with other pathogens, MABC causes the most rapid decline of lung function in CF patients, often resulting in early end-stage lung disease (Qvist et al., 2016). To current knowledge, MABC is the only human-transmissible NTM (Bryant et al., 2016). Recommended long-term multidrug treatment regimens for *M. abscessus* pulmonary disease (MAPD) harbor potential for numerous serious side effects and treatment outcome is often unfavorable (Daley et al., 2020). Early detection and treatment appear to improve the outcome (Qvist et al., 2016; Eikani et al., 2018). The diagnosis of MAPD is based on clinical, radiological, and microbiological criteria provided by the American Thoracic Society (Griffith et al., 2007). Diagnosis of MAPD in CF is particularly challenging, as MAPD overlaps in clinical and radiological presentation with CF lung disease. Additionally, the detection of MABC in the microbiological laboratory relies on cultural methods of sputum or bronchial aspirates/lavages. The abundance of more rapidly growing bacteria colonizing the respiratory tract of CF patients, e.g., *Pseudomonas aeruginosa*, frequently leads to overgrowth of mycobacterial cultures (Griffith et al., 2007).

While immunological diagnosis of tuberculosis is routinely performed worldwide using interferon (IFN)-γ-release assays (IGRAs) (Andersen et al., 2000), similar diagnostic approaches for MAPD are not yet established. In our own previous studies, we investigated a comparative T-cell-based immunologic approach for the detection and immune characterization of MABC infection in CF patients (Steindor et al., 2015). In the flow cytometric assays of this study, T cells of MABC patients showed stronger IFN-γ responses to *M. abscessus*-purified protein derivatives (PPDs) than to PPDs of Mycobacterium *tuberculosis* or *M. avium*. Despite significant cross-reactivity to the different PPDs, the relatively stronger T cell response of MABC patients to MABC PPD indicated the presence of specific antigenic targets for MABC. We thus aimed to identify abundantly expressed MABC-specific proteins with immunogenic properties. We performed proteome analyses of culture supernatants and bacterial lysates of different MABC strains. We found 12 mycobacterial proteins species specific for MABC with promising *in silico* evaluation for immunogenicity, rendering those candidates for subsequent immunological assays (Steindor et al., 2018). In the current study, we aimed to establish an MABC-specific IGRA with a subset of these proteins to evaluate host IFN-γ-response as a biomarker of MABC infection and potential diagnostic tool.

## Materials and Methods

### Study Subjects and Design

Study subjects were recruited at the pediatric or adult pulmonology departments of the University Hospital Essen (Department of Pediatric Pulmonology and Sleep Medicine and Department of Pulmonary Medicine, Ruhrlandklinik, Essen, Germany, respectively). Subjects with at least two separate sputum samples or one BAL sample positive for MABC prior study enrollment were enrolled in the *MABC-positive* group, including CF and non-CF subjects. Control groups were as follows: (1) CF subjects without microbiological evidence for NTM infection (no prior NTM infection with at least one negative sputum sample for NTM in the year prior to study enrollment). (2) Subjects with or without CF with NTM infection other than MABC (with the same NTM species recovered from at least two separate sputum samples or one biopsy prior to study enrollment). (3) Subjects with *M. tuberculosis*-IGRA confirmed current or prior tuberculosis (TB).

### Ethics Statement

The study was reviewed and approved by the Ethics Committee of the University of Duisburg-Essen (reference 18-8322-BO). The participants or their legal guardians provided their written informed consent to participate in this study.

### Purification of MABC Proteins

Five out of 12 putatively immunogenic MABC-specific protein candidates identified in our own previous studies (Steindor et al., 2018) were selected for purification and subsequent evaluation in this study, graded by their predicted number of T-cell epitopes (desired high) and structural homology to other proteins (desired low), namely, MAB_3249, MAB_0405c, MAB_0974, MAB_1614, and MAB_3801c. Proteins were purchased from ProteoGenix (Schiltigheim, France)^1^ where—in brief—the following purification approach was applied: protein cDNA sequences were cloned in pT7 expression vectors and proteins were overexpressed in *Escherichia coli* after protocol optimization for each protein regarding induction strategy, temperature/time for induction, choice of *E. coli* expression strain, and extraction condition (native or denatured with 8 M urea). Proteins were purified using polyhistidine(His)-Tag binding on nickel or glutathione *S*-transferase (GST)-Tag on glutathione sepharose resin. When purified as denatured protein extract, refolding was performed by stepwise dialysis against decreasing concentrations of urea (PBS with 4, 2, 1, and 0 M of urea, respectively). Protein purity was determined on 2D-SDS-PAGE with Coomassie blue staining. Endotoxin removal was performed on the final protein samples by Triton X-114 phase partition method (Triton^TM^ X-114, Sigma-Aldrich, Taufkirchen, Germany). Endotoxin levels were determined using a chromogenic limulus amebocyte lysate endotoxin assay kit (*Genscript ToxinSensor*^TM^ *Chromogenic LAL Endotoxin Assay Kit#L00350*). Proteins were stored and shipped in PBS buffer and frozen at -70°C until used for stimulation in ELISpot assays.

### ELISpot Assays

Twenty milliliters of heparinized blood was collected from each study subject, and peripheral blood mononuclear cells (PBMC) were separated by Ficoll gradient centrifugation. Numbers of PBMC were determined by an automated hematology analyzer (XP 300, Sysmex, Norderstedt, Germany). Various numbers of freshly isolated PBMC were grown without and with the four *M. abscessus* proteins MAB_3801c, MAB_0405c, MAB_3249, and MAB_1614 (0.1–50 μg/ml) for 1–3 days in order to define optimal cell culture conditions. Cell cultures stimulated with phytohemagglutinin (1 μg/ml) served as positive controls. PBMC were incubated in 200 μl AIMV medium (Gibco, Grand Island, NY, United States) at 37°C. As a first step, the cells were preincubated overnight in U bottom plates (BD Falcon, Nijmegen, Netherlands). In a comparative experiment, this first step was omitted (without preincubation). Thereafter, the cells were incubated for further 20 h in precoated ELISpot plates, using a standardized system to detect IFN-γ production (T-Track^®^ ELISpot kit, Lophius Biosciences, Regensburg, Germany). After this initial validation series, 300,000 PBMC were grown with 0.5–5 μg/ml protein for 2 days (1 day preincubation and 20 h incubation in ELISpot plates), because these conditions were considered optimal. Spot numbers were analyzed by an ELISpot reader (AID FluoroSpot, Autoimmun, Diagnostika GmbH, Strassberg, Germany). For the validation series, *M. abscessus*-specific results for each protein concentration and negative (unstimulated) controls are shown separately. Under the optimized conditions, we used 0.5, 1, and 5 μg/ml of *M. abscessus* proteins and considered the median response of these three cell cultures for further analysis. Negative (unstimulated) controls were subtracted from those of the *M. abscessus*-stimulated cultures and ELISpot results and given as spot increments.

In parallel, we tested for immunity against bacteria of the *M. tuberculosis complex* (*M. tuberculosis*, *Mycobacterium africanum, Mycobacterium bovis*, or *Mycobacterium leprae*), also responding to several environmental mycobacteria (e.g., *Mycobacterium kansasii, Mycobacterium marinum*, or *Mycobacterium szulgai*) (Andersen et al., 2000), using a commercially available ELISpot (T-SPOT.*TB*, Oxford ImmunoTech, Abingdon, United Kingdom). We exactly followed the manufacturer’s instructions. In brief, 250,000 PBMC per culture were stimulated for 20 h at 37°C with AIMV medium (negative control), phytohemagglutinin (positive control), or *M. tuberculosis*-specific peptide pools [early secretory antigenic target (ESAT-6) and culture filtrate protein-10 (CFP-10)] in a total volume of 150 ml. Reactions of ≥6 spots increment were considered positive.

### Bioinformatic Analyses and Strain Whole-Genome Sequencing

The proteins used in the assay were blasted (Camacho et al., 2009) against genomes of most pathogenic mycobacteria in former studies to confirm species specificity for MABC; their expression was confirmed in clinical MABC strains and the MABC reference strain ATCC 19977 by proteome analysis (Steindor et al., 2018). For NTM species occurring in this study with unknown expression of the study proteins, an additional BlastP search was performed with the study proteins against the NCBI database comprising non-redundant protein sequences. To verify the presence of the coding genes for the study proteins in MABC strains of MABC-positive patients with negative study assay results, respective MABC strains of the three first occurring discrepant cases during the study period were sequenced exemplarily. In brief, from genomic DNA extracted by the cetyltrimethylammonium bromide-(CTAB)-chloroform method (De Almeida et al., 2013), next-generation sequencing libraries were generated using a modified Illumina NextEra library kit protocol (Baym et al., 2015). Libraries were sequenced in a 2 × 150-bp paired end run on an Illumina NextSeq 500 instrument (Illumina, San Diego, CA, United States). Assemblies were made using Shovill v1.1.0 with trimming and downsampling to 100× coverage where applicable, and using SPAdes without read error correction as the assembly algorithm. Gene extraction/detection was performed using BioNumerics v7.6.

### Statistical Analysis

Cellular *in vitro* responses in patients with and without previous *M. abscessus* infection were compared by Mann–Whitney *U* test and responses in the four patient groups (including three control groups) by one-way ANOVA with Tukey’s multiple comparisons test, using GraphPad Prism version 8.0.1 (GraphPad Software, San Diego, CA, United States).

## Results

### Subject Enrollment and Characteristics

We enrolled 47 subjects in total, of which 12 had confirmed MABC infection (group 1), 17 had CF without any mycobacterial infection (group 2), nine had NTM infection other than MABC (group 3), and nine had TB (group 4). Study subjects’ characteristics are summarized in Table 1. NTM species of the NTM group comprised members of the *M. avium* complex (MAC) (*n* = 7), *Mycobacterium canariasense*, *Mycobacterium malmoense* (*n* = 1 each, both in a single patient), and *Mycobacterium simiae* (*n* = 1). In the TB group, all patients had cultural detection of *M. tuberculosis* except one *M. africanum*. One TB patient had a *M. malmoense* NTM coinfection.

**TABLE 1:** Characteristics of study subjects in total and divided by study groups are shown.

	**Total**	**MABC**	**CF**	**NTM^a^**	**TB^b^**
*n*	47	12	17	9	9
CF (*n*)	31	10	17	5	0
(%)	66.0%	83.3%	100.0%	55.6%	0.0%
Male sex (*n*)	26	8	9	3	6
(%)	55.3%	66.7%	52.9%	33.3%	66.7%
Age					
Median	20	24	18	44	15
IQR	19.5	16	17	22	8
ppFEV1					
Median	70	67	68	56	98
IQR	47	29.5	42	35	9

*MABC, subjects with M. abscessus complex infection; CF, subjects with cystic fibrosis without evidence for any mycobacterial infection; NTM, subjects with mycobacterial infections other than M. abscessus or M. tuberculosis complex; TB, subjects with M. tuberculosis complex infection. ^a^NTM species comprised M. avium complex (n = 7), M. malmoense, M. simiae, and M. canariasense (n = 1 each). ^b^One TB patient had coinfection with M. malmoense.*

### Purification of MABC Proteins

After individual purification protocol optimization for each protein, all proteins except MAB_0974 were purified by affinity vs. His-Tag. Affinity vs. GST-Tag was used for MAB_0974. All proteins except MAB_3249 were obtained from native protein extract. After extraction from denatured protein extract, MAB_3249 was successfully refolded by dialysis. Final protein purity and endotoxin levels after purification are given in Table 2. Final western blot results of the purified proteins are shown in Supplementary Figure 1. Due to the low achieved purity of MAB_0974, the protein was omitted from further investigations and endotoxin removal was not performed.

**TABLE 2:** Characteristics of study proteins.

**Protein**	**Length (AA)**	**PDB ID of closest protein homolog (probability)**	**Putative function**	**Predicted MHC-II high binders**	**Purity (%)**	**Endotoxin levels (EU/ml)**
MAB_3249	147	4V4N_BT (42.36%)	Ribosomal preprotein translocase subunit	92	>95	2
MAB_0405c	195	3K7C_D (98.82%)	NTF2-like transpeptidase	58	>95	5
MAB_1614	213	3FKA_B (98.58%)	NTF2-like protein	70	>95	12.9
MAB_3801c	179	4QTQ_A (98.76%)	Unknown	36	>95	1.2
MAB_0974	606	5Z0R_A (18.56%)	Extracellular solute-binding	75	30	n/a

*The length of each protein is given in number of amino acids. Data on putative function of each protein assumed by its closest known structural homolog (presented as protein data bank identifier) with estimated probability of analogy and the estimated number of MHC-II high binders were taken from our previous publication (Steindor et al., 2018). Purity and endotoxin levels are shown. No endotoxin levels were determined for MAB_0974, as it was omitted from further analysis due to poor purification results.*

### Optimization of Cell Culture Conditions

**FIGURE 1:**
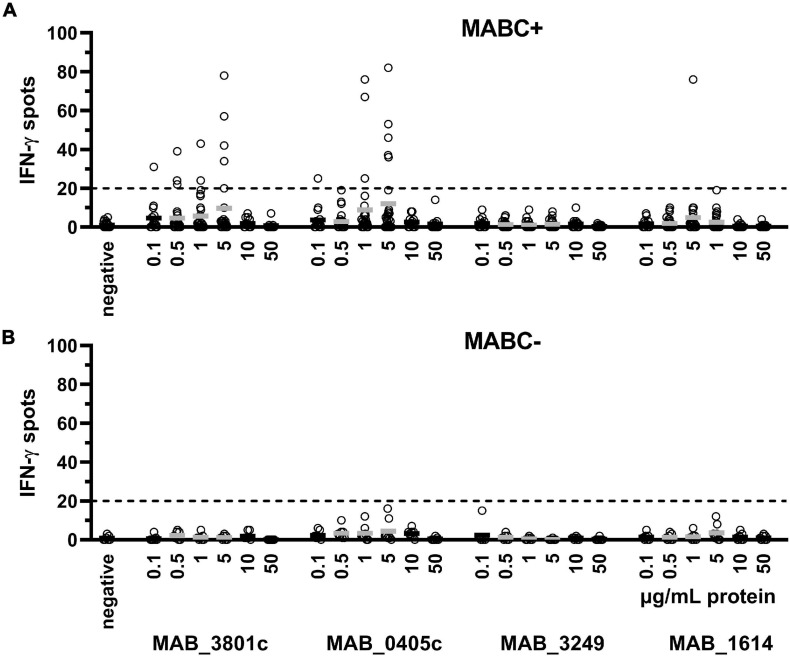
PBMC were grown with six different concentrations of each of the four *M. abscessus* proteins MAB_3801c, MAB_0405c, MAB_3249, and MAB_1614 (0.1–50 μg/ml), and IFN-γ production was determined by ELISpot. We analyzed cellular responses in patients with previous culture-confirmed *M. abscessus* infection (MABC+, *n* = 14–28 experiments, **A**) and without *M. abscessus* infection (MABC-, *n* = 8 experiments, **B**). Mean values are shown by the solid horizontal lines; gray labeling indicates optimal concentrations. In the validation series, none of the MABC-negative patients showed more than 20 IFN-γ spots (dotted line). This analysis includes all datasets of the validation experiments, applying 200,000, 300,000, and 400,000 PBMC and cell cultures without preincubation and with 1 and 2 days preincubation. Of note, the subsequent figures only contain results using the optimized cell culture conditions (300,000 PBMC and 1 day preincubation).

To optimize the assay conditions, we used six different concentrations of each of the four *M. abscessus* proteins (0.1–50 μg/ml). IFN-γ production showed a dose dependency, reaching a maximum at intermediate protein concentrations of 0.5–5 μg/ml protein, which was observed in patients with and without prior culture-confirmed *M. abscessus* infection (Figure 1). Of note, the proteins MAB_3801c and MAB_0405c induced the strongest IFN-γ response, whereas the response to MAB_3249 was in the range of negative controls and the response to MsAB_1614 was weak.

Stimulation of various cell numbers (200,000, 300,000, and 400,000 PBMC per cell culture) identified a number of 300,000 PBMC to show the best performance in discriminating between MABC-infected subjects and MABC-negative controls (Supplementary Figure 2). Specific IFN-γ production of 200,000 appeared as too weak, and negative controls showed considerable numbers of spots when growing 400,000 PBMC (mean 3 spots, range 0–16).

In order to increase the sensitivity (by allowing close contact between the cells), PBMC were preincubated in U bottom plates for 1 and 2 days, respectively. Thereafter, cells were grown for 20 h in flat-bottom ELISpot plates. The comparative analysis of cells with and without preincubation showed that preincubation in U bottom plates increased the number of spots (Supplementary Figure 3). Overall, the results after 1 and 2 days of preincubation were similar. However, negative controls showed more than twofold higher spot increments after 2 days of preincubation. We therefore decided to use a 1-day preincubation period, i.e., a total culture duration of 2 days.

### Performance of MABC-Specific IGRA for Detection of MABC Infection

**FIGURE 2:**
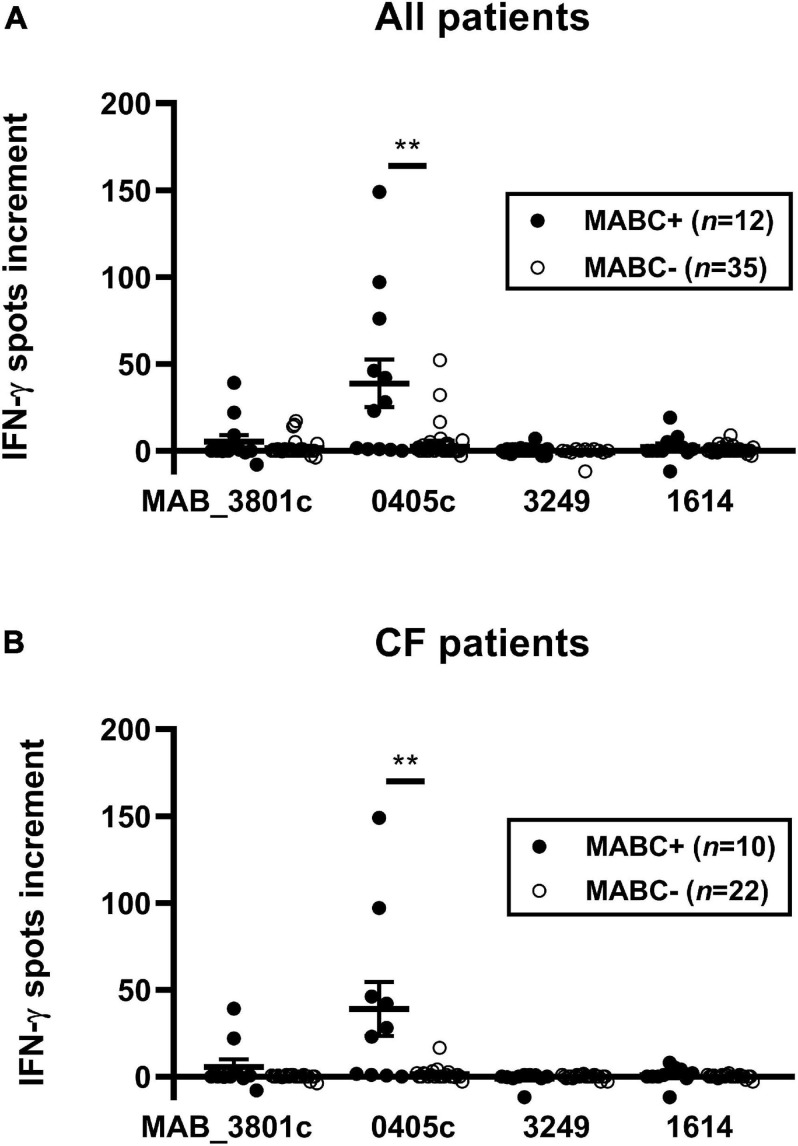
PBMC of patients with and without *M. abscessus* (MABC) infection were stimulated with the four *M. abscessus* proteins MAB_3801c, MAB_0405c, MAB_3249, and MAB_1614, and IFN-γ production was determined by ELISpot. We analyzed cellular responses in all 47 patients **(A)** and separately in 32 patients with cystic fibrosis (CF) **(B)**, using the optimized cell culture conditions, as defined in Section “Materials and Methods.” Data are indicated as mean and standard error of the mean (SEM). Patients with (+) and without (-) MABC infection were compared by Mann–Whitney *U* test. Cellular *in vitro* responses are given as spots increment, i.e., MABC protein stimulated minus negative control. **p < 0.01.

We compared IFN-γ responses of 12 subjects with established diagnosis of MABC infection (10 CF and two non-CF) with 35 controls using optimized cell culture conditions. Controls comprised three groups, (1) CF subjects without mycobacterial infection, (2) subjects with NTM infection other than MABC, and (3) subjects with TB. Cellular *in vitro* responses toward the *M. abscessus* protein MAB_0405c were stronger in the MABC group than in the control groups (all patients: 39 vs. 4 spots per 300,000 PBMC, *p* = 0.004; CF patients only: 39 spots vs. 1 spot per 300,000 PBMC, *p* = 0.003; data represent mean values) (Figure 2). Furthermore, cellular responses toward the protein MAB_3801c tended to be stronger in patients with vs. without MABC infection, whereas responses toward MAB_3249 and MAB_1614 were nearly undetectable in both groups. Notably, positive controls with phytohemagglutinin exceeded 40 spots in all assay runs, indicating that all results were valid.

**FIGURE 3:**
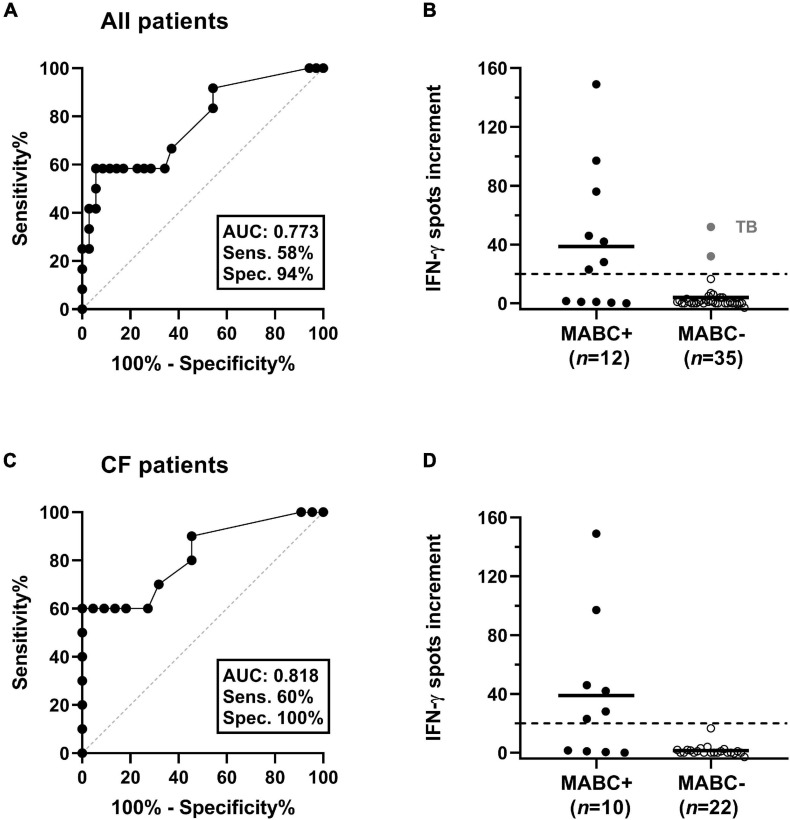
Discrimination between patients with and without *M. abscessus* (MABC) infection. This analysis considered either all patients **(A,B)** or only patients with cystic fibrosis (CF) **(C,D)**. **(A,C)** Results of receiver operating characteristic (ROC) curve analyses. It was analyzed if ELISpot responses to the MAB_0405c protein were predictive of (culture-confirmed) MABC infection. **(B,D)** Responses of the MAB_0405c ELISpot are compared in patients with and without MABC infection (Mann–Whitney *U* test). Positive results in two patients with *M. tuberculosis* infection (TB) are shown as gray filled circles. The horizontal lines indicate the mean values, and the dashed lines indicate the cut-off value as defined by the ROC curve analysis (20 spots increment).

Receiver operating characteristic (ROC) curve analysis indicated that at least 20 spot increments in the MAB_0405c ELISpot were highly predictive of MABC infection (Figure 3). Considering all patients, the sensitivity was 58% and specificity 94% (area under curve 0.773). In CF patients, the sensitivity was 60% and specificity 100% (area under curve 0.818).

**FIGURE 4:**
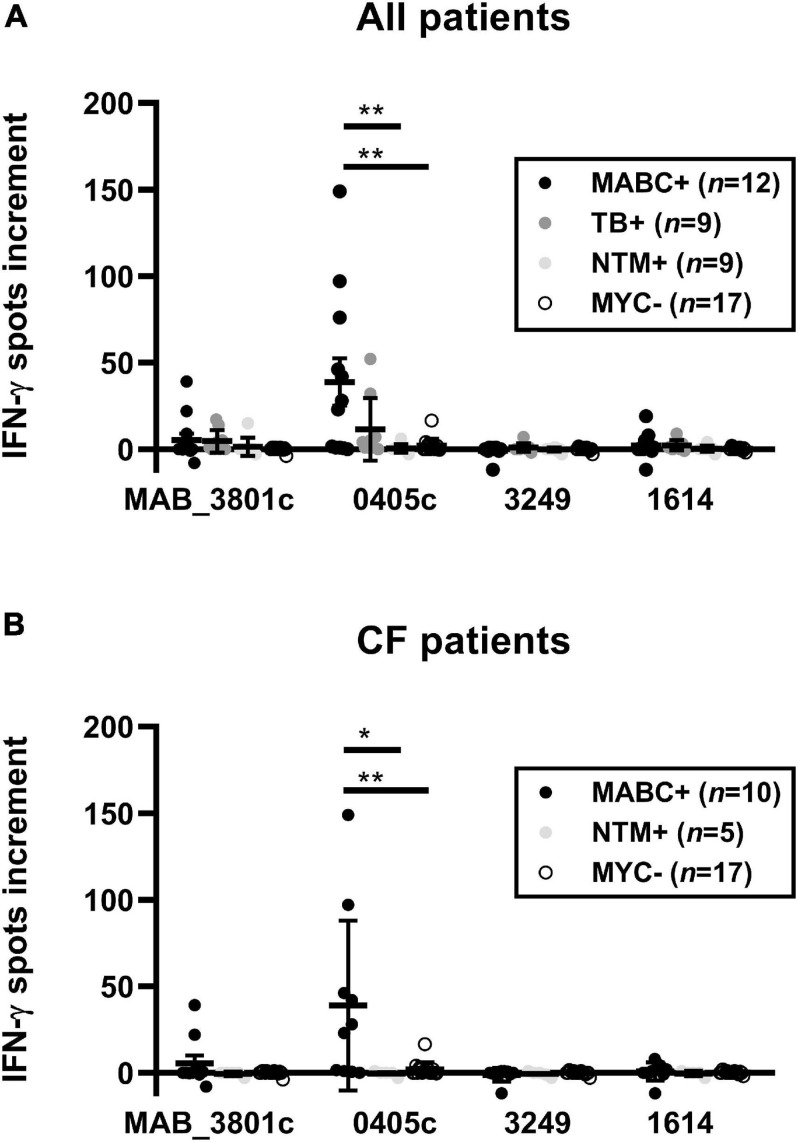
PBMC of patients with *M. abscessus* (MABC) infection, subjects with cystic fibrosis (CF) without mycobacterial infection (MYC-), subjects with NTM infection other than MABC (NTM), and subjects with tuberculosis (TB) were stimulated with the four *M. abscessus* proteins MAB_3801c, MAB_0405c, MAB_3249, and MAB_1614, and IFN-γ production was determined by ELISpot. We analyzed cellular responses in all 47 patients **(A)** and separately in 32 patients with cystic fibrosis (CF) **(B)**, using the optimized cell culture conditions, as defined in Section “Materials and Methods.” Data are indicated as mean and standard error of the mean (SEM). The four patient groups were compared by one-way ANOVA. Cellular *in vitro* responses are given as spots increment, i.e., MABC protein stimulated minus negative control. *p < 0.05; **p < 0.01.

A detailed analysis of the various control groups showed that two out of nine patients with TB also responded to MAB_0405c, showing 32 and 52 spot increments, respectively (Figures 3, 4). These two TB patients both had no known NTM coinfection. Of note, in all TB patients, the diagnosis was confirmed by ELISpot (T-SPOT.*TB*, Oxford Immunotec, Abingdon, Oxfordshire, United Kingdom).

### Bioinformatic Analyses and Strain Sequencing

BlastP searches did not yield significant hits for the study proteins MAB_3801c, MAB_0405c, MAB_3249, and MAB_1614 in formerly uninvestigated NTM occurring in the study (i.e., *M. simiae*, *M. malmoense*, *M. canariasense*, data not shown). Whole-genome sequencing was performed on cultured MABC isolates of three MABC-positive study subjects with negative assay results (subjects 1, 2, and 3, respectively, see Table 3). All coding sequences of the four study proteins were found in all three strains with a 100% gene coverage (full length, no indels) and at least 99% similarity toward the reference allele from *M. abscessus* strain ATCC19977T, excluding lack of genomic protein coding as a cause of negative assay results. Genome sequences are accessible *via* NCBI sequence read archive^2^ under the accession number PRJEB44160.

**TABLE 3:** Subject characteristics of *M. abscessus* complex positive subjects.

**Subject**	**Assay result^*a*^**	**Sex**	**Age in years**	**ppFEV1**	**Disease**	**MABC subspecies**	**Colony morphotype**	**First MABC isolation prior enrollment in months**	**Last MABC isolation prior enrollment in months**	**MAPD**	**MABC treatment**	**MABC clearance**
1	0	F	16	63	CF	*abscessus*	n/a	15	12	+	+	+
2	0.5	F	16	57	CF	*abscessus*	Rough	36	Persistent	+	+	–
3	1.5	F	32	77	CF	*abscessus*	Rough	14	Persistent	–	–	–
4	46	M	25	100	CF	*abscessus*	n/a	19	Persistent	–	–	–
5	23	M	36	63	CF	*abscessus*	Smooth	16	Persistent	+	+	–
6	1	M	32	71	CF	*abscessus*^b^	Smooth	10	Persistent	–	–	–
7	76	M	20	25	PCD	n/a	n/a	84	72	–	–	+
8	28	M	26	30	CF	*abscessus*	Rough	22	Persistent	+	+	–
9	42	M	15	92	CF	*abscessus*	Smooth	7	Persistent	–	–	–
10	149	M	12	78	CF	*abscessus*	Rough	9	9	+	+	+
11	97	M	23	109	CF	*massiliense*	Rough	6	Persistent	–	–	–
12	1	F	49	37	Asthma, breast cancer	*massiliense*	Smooth	2	Persistent	+	+	–

*n/a, not available; CF, cystic fibrosis; PCD, primary ciliary dyskinesia; MABC, Mycobacterium abscessus complex; MAPD, Mycobacterium abscessus pulmonary disease. ^a^Median spot increment, cut-off ≥20 spots. ^b^Coinfection with M. avium complex.*

## Discussion

While a central role of IFN-γ in the host immune response to MABC has been shown, its diagnostic potential is not exploited yet (Jeon et al., 2009; Steindor et al., 2015). In this study, we aimed to establish a species-specific IGRA for MABC. Four species-specific MABC proteins (i.e., MAB_3801c, MAB_0405c, MAB_3249, and MAB_1614) were tested in MABC-positive subjects and three control groups (i.e., CF subjects without mycobacterial infection, subjects with NTM infection, and subjects with TB). Upon stimulation with MAB_3801c and MAB_0405c, MABC patients showed higher IFN-γ responses compared with controls. Stimulation with MAB_3249 and MAB_1614 did not induce relevant IFN-γ immune responses in either group. Strongest IFN-γ responses and best discrimination of MABC patients from controls were achieved upon stimulation with MAB_0405c with an overall diagnostic sensitivity and specificity of 58 and 94%, respectively. In CF patients, diagnostic sensitivity and specificity were even higher with 60 and 100%, respectively. MAB_0405c is a small protein of 195 amino acids in length with unknown function and closest structural homology to an NTF2-like transpeptidase of *Campylobacter jejuni* (Steindor et al., 2018). We show that a majority of MABC infections may be identified by this assay upon stimulation with MAB_0405c. However, five out of 12 MABC-positive subjects (40%) had negative test results, of whom three had clinical and radiological MAPD. Interestingly, all female MABC-positive subjects enrolled in this study showed no MABC-specific IFN-γ response. We speculate that this might represent a sample size bias rather than a biologically significant finding as a significant sex dependency of IGRAs is not described (Pai et al., 2014). Both pathogen and host factors might cause the relatively low sensitivity of the assay. The lack of genomic presence of protein coding sequences of the assay proteins by the MABC strains was ruled out by whole-genome sequencing in three assay-negative cases. Immune evasion of the pathogen, e.g., by the capability of MABC to mask immunogenic surface proteins with glycopeptolipids, has been shown to be a possible mechanism for explaining negative IGRA results in these patients (Rhoades et al., 2009). However, in two assay-negative cases, the strains grew in rough colony morphotypes, which are characterized by the loss of glycopeptolipid expression and usually represent invasive, immunogenic bacterial phenotypes, rendering this mechanism of immune evasion unlikely in these cases (Pawlik et al., 2013). Overall, (additional) host factors seem likely to cause the low or missing IFN-γ response in a majority of assay-negative cases, yet the underlying mechanisms remain speculative. Antigenicity of MAB_0405c is probable to differ between individuals due to variable processing of mycobacterial T cell antigens (Boom et al., 2003). Additionally, the time point of IFN-γ response manifestation and its duration during the course of MABC infection remain unclear. When extrapolating from TB, we would expect IFN-γ response to occur within several weeks postinfection and persist on a certain level afterward in most cases, even after clearing of the infection (Pai et al., 2006; Chee et al., 2007; Mack et al., 2009). The value of longitudinal assessment of IFN-γ response in TB remains controversial and is entirely uninvestigated for MAPD. Our cross-sectional study with a small sample size is unfit to associate of MAPD patient characteristics to the assay results or to give definite answers to questions on the longitudinal course of immune response. However, three formerly MABC-infected subjects (MABC subjects 1, 7, and 10, respectively; see Table 3) had positive assay results despite apparent clearance of the infection (up to 72 months) before study enrollment, indicating a persisting IFN-γ response after clearance of MABC—or a latent-state MABC infection.

Finally, the presented assay supports diagnostics of MAPD, especially in the absence of respiratory specimen (e.g., children) or in cases of (repetitive) overgrowth of mycobacterial sputum cultures by other microbes. Both settings are frequently encountered in the context of CF, in which the assay has proven particularly accurate. The assay represents an additional, culture-independent tool of disease identification and characterization and might, for example, lead to targeted MAPD diagnostics (e.g., bronchoalveolar lavage and high-resolution computed tomography) for timely detection of MAPD, which has proven beneficial toward the disease outcome (Qvist et al., 2016; Ravnholt et al., 2018). The use of additional proteins or peptides for blood stimulation might improve the assay in the future, but those peptides are still to be determined. Future studies should also target larger cohorts of MAPD patients to evaluate the merit of this assay for disease characterization and longitudinal assessment of MABC infection.

## Data Availability Statement

The genome sequencing data presented in this study can be found in online repositories. The names of the repository/repositories and accession number(s) can be found below: https://www.ncbi.nlm.nih.gov/bioproject/PRJEB44160/, accession: PRJEB44160.

## Ethics Statement

The studies involving human participants were reviewed and approved by the Ethics Committee of the University of Duisburg Essen (reference 18-8322-BO). Written informed consent to participate in this study was provided by the participants or their legal guardian/next of kin.

## Author Contributions

MS and ML conceived and designed the study, put all study data together, and drafted the manuscript with substantial contributions from all authors. MS raised the funding. MS, FS, MO, SvS, MW, JK, and SiS collected study specimen and collected and interpreted clinical data. ML performed and analyzed the ELISpot experiments with contribution from PH. MD and FM performed the genomic analyses. ML performed the statistical analyses. All authors listed have made a substantial, direct and intellectual contribution to the work, and approved it for publication.

## Conflict of Interest

The authors declare that the research was conducted in the absence of any commercial or financial relationships that could be construed as a potential conflict of interest.

## Footnotes

1 https://www.proteogenix.science/

2 https://www.ncbi.nlm.nih.gov/sra

## Supplementary Material

Supplementary Figure 1 Western blots (reduced PAGE with Coomassie blue staining) of the final purified protein samples of the study. Due to the low achieved purity of MAB_0974, the protein was omitted from further investigations.

Supplementary Figure 2 Various numbers of PBMC (200,000–400,000) **(A–C)** were grown with six different concentrations of each of the four *M. abscessus* proteins MAB_3801c, MAB_0405c, MAB_3249, and MAB_1614 (0.1–50 μg/ml) and IFN-γ production was determined by ELISpot. We here combined data in patients with and without previous culture-confirmed *M. abscessus* infection. Mean values are shown by the solid horizontal lines; gray labeling indicates optimal concentrations. The dotted line shows the cut-off for positivity, as determined by the ROC curve analysis.

Supplementary Figure 3 PBMC were grown for 1–3 days, comprising a preincubation period of 0–2 days **(A–C)**. We used six different concentrations of each of the four *M. abscessus* proteins MAB_3801c, MAB_0405c, MAB_3249, and MAB_1614 (0.1–50 μg/ml), and IFN-γ production was determined by ELISpot. We here combined data in patients with and without previous culture-confirmed *M. abscessus* infection. Mean values are shown by the solid horizontal lines; gray labeling indicates optimal concentrations. The dotted line shows the cut-off for positivity, as determined by the ROC curve analysis.
