# Supplementary figures and images for: Species-Specific Interferon-Gamma Release Assay for the Diagnosis of Mycobacterium abscessus Complex Infection
Source: Front Microbiol. 2021 Jul 12;12:692395. doi: 10.3389/fmicb.2021.692395 (PMC8312262; doi:10.3389/fmicb.2021.692395)

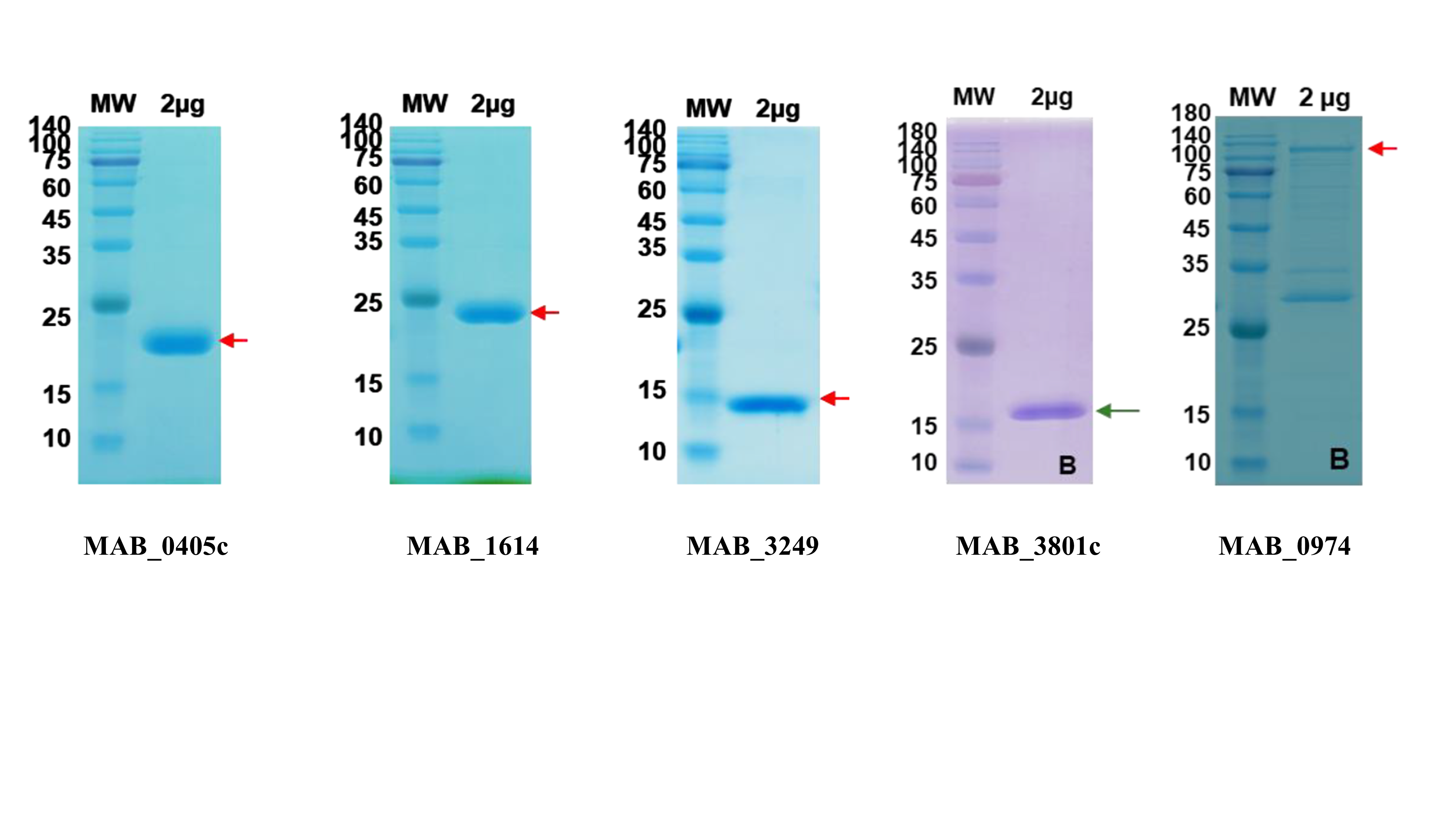

Supplement: Supplementary Figure 1 — Western blots (reduced PAGE with Coomassie blue staining) of the final purified protein samples of the study. Due to the low achieved purity of MAB_0974, the protein was omitted from further investigations. [file Image_1.PNG]
