# Supplementary material for: Species-Specific Interferon-Gamma Release Assay for the Diagnosis of Mycobacterium abscessus Complex Infection
Source: Front Microbiol. 2021 Jul 12;12:692395. doi: 10.3389/fmicb.2021.692395 (PMC8312262; doi:10.3389/fmicb.2021.692395)
Supplement: Supplementary file 4 [file Table_1.DOCX]

| **Subject** | **Test result** | **Study group** | **Mycobacterial species** | **Disease** | **Sex** | **Age** | **ppFEV1** |
| --- | --- | --- | --- | --- | --- | --- | --- |
| 1 | 0 | MABC | M. abscessus | CF | F | 16 | 63 |
| 2 | 0.5 | MABC | M. abscessus | CF | F | 16 | 57 |
| 3 | 1.5 | MABC | M. abscessus | CF | F | 32 | 77 |
| 4 | 46 | MABC | M. abscessus | CF | M | 25 | 100 |
| 5 | 23 | MABC | M. abscessus | CF | M | 36 | 63 |
| 6 | 1 | MABC | M. abscessus | CF | M | 32 | 71 |
| 7 | 76 | MABC | Unspecified MABC | PCD | M | 20 | 25 |
| 8 | 28 | MABC | M. abscessus | CF | M | 26 | 30 |
| 9 | 42 | MABC | M. abscessus | CF | M | 15 | 92 |
| 10 | 149 | MABC | M. abscessus | CF | M | 12 | 78 |
| 11 | 97 | MABC | M. massiliense | CF | M | 23 | 109 |
| 12 | 1 | MABC | M. massiliense | asthma, breast cancer | F | 49 | 37 |
| 13 | 0 | NTM | MAC | CF | F | 44 | 29 |
| 14 | 0 | NTM | MAC | CF | F | 33 | 35 |
| 15 | 1 | NTM | MAC | COPD | M | 57 | 20 |
| 16 | 0 | NTM | MAC | non-CF bronchiectasis | M | 81 | 104 |
| 17 | 0 | NTM | M. canariasense, M. malmoense | IFN-γ receptor deficiency | M | 8 | 98 |
| 18 | -1 | NTM | MAC | CF | F | 24 | 56 |
| 19 | 0 | NTM | M. simiae | asthma, ABPA | F | 56 | 70 |
| 20 | -3 | NTM | MAC | CF | F | 55 | 44 |
| 21 | 1 | NTM | MAC | CF | F | 9 | 69 |
| 22 | 52 | TB | M. tuberculosis | TB | M | 6 | 97 |
| 23 | 32 | TB | M. tuberculosis | TB | F | 16 | 107 |
| 24 | 1 | TB | M. tuberculosis | TB | F | 12 | 105 |
| 25 | -2 | TB | M. africanum | TB | M | 33 | n/a |
| 26 | 0 | TB | M. tuberculosis | TB | M | 20 | n/a |
| 27 | 0 | TB | M. tuberculosis | TB | M | 12 | 101 |
| 28 | 7 | TB | M. tuberculosis | TB | M | 7 | 91 |
| 29 | 1 | TB | M. malmoense | latent TB, autoimmune hepatitis | F | 56 | 79 |
| 30 | 1.5 | CF | - | CF | M | 17 | 36 |
| 31 | 0 | CF | - | CF | F | 18 | 41 |
| 32 | -1 | CF | - | CF | F | 15 | 92 |
| 33 | 0 | CF | - | CF | F | 15 | 49 |
| 34 | 0 | CF | - | CF | M | 73 | 35 |
| 35 | 1 | CF | - | CF | M | 38 | 39 |
| 36 | 4 | CF | - | CF | M | 15 | 98 |
| 37 | 1 | CF | - | CF | M | 18 | 68 |
| 38 | 0 | CF | - | CF | M | 17 | 101 |
| 39 | 0 | CF | - | CF | F | 38 | 50 |
| 40 | 3 | CF | - | CF | F | 16 | 78 |
| 41 | 0 | CF | - | CF | F | 11 | 79 |
| 42 | 0 | CF | - | CF | F | 26 | 88 |
| 43 | 4 | CF | - | CF | M | 33 | 88 |
| 44 | 0 | CF | - | CF | M | 12 | 45 |
| 45 | 0 | CF | - | CF | M | 59 | 87 |
| 46 | 2 | CF | - | CF | M | 20 | 68 |
| 46 | 0 | CF | - | CF | F | 25 | 51 |

Table S1 Study subjects with characteristics and study test result (median spot increment, cut-off ≥20 spots). *M. abscessus* subspecies is given if not otherwise indicated. MABC *M. abscessus* complex; MAC *M. avium* complex. CF Cystic Fibrosis, NTM non-tuberculous mycobacteria; TB Tuberculosis; PCD Primary Ciliary Dyskinesia; COPD Chronic Obstructive Pulmonary Disease; ABPA Allergic Bronchopulmonary Aspergillosis
